# Capsule Enteroscopy Using the Mirocam^®^ versus OMOM^®^ Systems: A Matched Case–Control Study

**DOI:** 10.3390/life13091809

**Published:** 2023-08-25

**Authors:** Maria Manuela Estevinho, Rolando Pinho, Adélia Rodrigues, Ana Ponte, João Correia, Pedro Mesquita, Teresa Freitas

**Affiliations:** 1 Department of Gastroenterology, Vila Nova de Gaia Espinho Hospital Center, 4434-502 Vila Nova de Gaia, Portugal; 2 Unit of Pharmacology and Therapeutics, Department of Biomedicine, Faculty of Medicine, University of Porto, 4200-319 Porto, Portugal

**Keywords:** small bowel capsule endoscopy, enteroscopy, gastrointestinal diseases

## Abstract

Although several devices are available for small bowel capsule endoscopy, few studies have compared their visualization quality and diagnostic yield, despite users reporting subjective differences between them. This study aims to compare two widely used systems (Mirocam^®^ MC1600 and OMOM^®^ HD). Patients who underwent OMOM^®^ HD capsule enteroscopy between August 2022 and February 2023 were prospectively included consecutively (cases). Controls were retrospectively selected from a database of patients who underwent Mirocam^®^ MC1600 enteroscopy between March 2018 and July 2022 in a 1:1 ratio. Controls were matched for potential confounders (age, sex, indication, hospitalization, comorbidities, and opioid prescription). The small bowel cleanliness (global and divided by tertiles), the diagnostic yield (positive findings) and the transit times (TT) were compared. Overall, 214 patients were included (107:107). Global bowel preparation was similar between the OMOM^®^ and Mirocam^®^ groups. However, the average scores for each tertile were significantly higher when the OMOM^®^ HD capsule was used (*p* < 0.05). Small bowel TT was shorter for OMOM^®^ HD (265 ± 118 versus 307 ± 87 min, *p* = 0.020), while the diagnostic yield (55.0%) and relative distribution of lesions were similar. This study suggests that capsule characteristics, namely resolution, and illumination, systematically interfere with the perception of preparation quality. However, this did not affect the diagnostic yield.

## 1. Introduction

Since its development back in 1997, small bowel capsule endoscopy (SBCE) has undergone significant progress, benefiting from technological breakthroughs that led to improved imaging quality, extended battery life, and enhancement of pathology recognition software [1,2]. SBCE is currently widespread, and, according to recent forecasts, the global market for capsule endoscopy is projected to expand at a compound annual growth rate of 12.1%, reaching a volume of USD 1.4 billion by 2028 [3].

Due to its excellent safety profile, patient tolerability, and the potential to visualize entire small bowel mucosa, SBCE is the primary approach for diagnosing various conditions, such as suspected small bowel bleeding, suspected or confirmed non-stenotic Crohn’s disease, small bowel tumors or polyps, and refractory celiac disease [4,5]. However, unlike conventional endoscopy, it is not possible to cleanse the bowel during the SBCE procedure [6]. Indeed, inadequate small bowel cleanliness and incomplete visualization, mostly due to battery timespan, are the key factors explaining the false negative rates of SBCE, which may reach 20% in the case of neoplastic diseases [7,8]. Therefore, ensuring adequate bowel visualization is crucial for enhancing the detection and monitoring of small bowel diseases. Currently, there is no agreement or consensus regarding the specific regimen and purging requirements that should be followed prior to SBCE [9]. Likewise, the impact of capsule specifications (such as resolution and light) on the ability to observe mucosa and retrieve a diagnosis remains unknown, particularly regarding more recent models. Notwithstanding, clinical experience suggests that the perception of small bowel cleanliness relies solely on the model used and not on bowel preparation itself.

Over time, numerous capsule systems have been developed. Nevertheless, only a limited number of studies (ten in total) have compared devices from different brands, with nine of them using PillCam as a reference [10]. It is worth noting that only a few have specifically compared models from PillCam^®^ with Mirocam^®^ MC1600 (n = 3) [11,12,13] or OMOM^®^ HD [14]. Even though these studies report no differences in the diagnostic yield, none have compared the perception of small bowel cleanliness.

In this study, we aimed to compare, for the first time, two systems frequently used to evaluate the small bowel (OMOM^®^ HD and Mirocam^®^ MC1600) regarding transit times, quality of visualization, and diagnostic yield in real-world practice. We decided to compare these systems because their price range is similar, being the choice between one versus the other based on subjective user experience aspects, such as the quality of visualization, for which no evidence is available.

## 2. Materials and Methods

### 2.1. Study Design

We performed a matched case–control study of adults aged ≥ 18 years submitted for capsule endoscopy in a tertiary hospital center (inclusion criteria). Case-control matching is a statistical technique that selects control subjects resembling the cases based on specific criteria. It aims to minimize confounding factors and improve comparability, resulting in more reliable and valid study outcomes.

Cases were consecutively selected from all patients who underwent OMOM^®^ HD capsule (Jinshan Science and Technology, Yubei, China) enteroscopy between August 2022 and February 2023 in our unit. Controls were retrospectively selected from a database of 306 patients who underwent small bowel examinations with the Mirocam^®^ MC1600 capsule (IntroMedic, Seoul, Republic of Korea) between March 2018 and July 2022 in a 1:1 ratio. Patients who were younger than 18 years old were removed from this analysis (exclusion criteria).

Controls were matched for potential confounding factors (age, sex, indication, setting (outpatient or inpatient), presence of relevant comorbidities, and use of opioids). The same preparation protocol (clear liquids on the previous day plus 2 L of KleanPrep^®^ between 7 p.m. and 9 p.m. on the day before and 2 L between 5 a.m. and 7 a.m. on the day of the examination) were used by both groups. The recorder was removed either after 12 h from capsule ingestion or earlier if real-time viewing confirmed that the device had reached the colon. Gastroenterologists with extensive expertise in SBCE (examination of over 350 procedures) analyzed all videos.

### 2.2. Data Collection

Anonymized patient data were collected, including patient demographic characteristics (gender and age), comorbidities (chronic heart, kidney or liver disease, diabetes mellitus, and abdominal surgeries), clinical presentation, and indication, as well as previous or consecutive endoscopic, radiologic, or surgical procedures.

Regarding small bowel findings, we assessed the detection of relevant findings throughout the small bowel (diagnostic yield, DY) and the presence of specific findings such as active bleeding, angioectasia, villous atrophy, erosions, ulcers, stenosis, and polyps/tumors. We also reported the presence of extra-small bowel findings. SBCE was considered complete when the colon was visualized during the procedure. Capsule retention was defined as the presence of a capsule in the small bowel for a duration exceeding two weeks or causing symptoms that required its early removal.

The level of cleanliness was recorded through the adapted Brotz scale [15,16]. This score comprises a quantitative and qualitative evaluation and assesses five parameters ((i) percentage of mucosa observed, (ii) presence of fluid and residues, (iii) bile/chyme staining, (iv) brightness, and (v) presence of bubbles), each scored from 0 (severe impairment) to 2 (minimal impairment) and was defined to globally evaluate the small bowel; a corresponding score ≥ 7/10 denoted adequate preparation. In this study, cleanliness was evaluated globally and per tertile (small bowel transit time divided by three-adapted Brotz scale).

### 2.3. Statistical Analysis

Descriptive analysis was conducted on the data, and the results are reported as mean ± standard deviation or as median with the interquartile range for continuous variables where appropriate, while categorical variables are presented as frequency and percentages. The statistical analysis was performed using IBM SPSS Statistics version 28 (IBM Corporation, Armonk, NY, USA), using the chi-square test (for categorical variables) and independent samples *t*-test (for continuous variables). 

## 3. Results

### 3.1. Patient Characterization

Over seven months (August 2022 and February 2023), 107 patients were submitted for capsule enteroscopy with the OMOM^®^ HD system (cases). Most of these were female (61.7%) and had a mean age of 54.0 ± 18.5 years old (Table 1). The indications for performing SBCE were mainly the presence of iron-deficiency anemia (57.9%), suspicion of Crohn’s disease (CD, 26.2%), or the monitoring of patients already diagnosed with CD (11.2%). 

The cohort of patients who performed SBCE between March 2018 and July 2022 using Mirocam^®^ MC1600 (n = 306) was used as a control. Considering that this cohort differed from the one using OMOM^®^ HD in several factors that could potentially act as confounders (such as the percentage of females (*p* = 0.041), presence of chronic heart disease (*p* = 0.038), and diabetes mellitus (*p* = 0.040)), as well as the indication for SBCE (47.1% for the investigation of iron-deficiency anemia compared to 58.9% in the OMOM^®^ HD group, *p* = 0.039) a matched case–control study was conducted. As such, controls were selected for cases in a 1:1 ratio, accounting for age, sex, indication, setting, presence of relevant comorbidities, and opioid use. The characteristics of the matched controls (n = 107) are presented in the fourth column of Table 1 and, as expected, do not differ significantly from the cases.

### 3.2. Capsule Completion and Transit Times

Only two cases of capsule retention occurred in patients with suspected CD (Table 2). Of the 214 patients included in the matched analysis, the device failed to reach the cecum in only four cases during the battery timespan. No differences between the capsule endoscopy systems were detected, and no procedure-related complications occurred.

Small bowel time was significantly inferior in the group that used the OMOM^®^ HD system (mean difference of 52 ± 14 min, *p* = 0.032), while no differences were detected for gastric transit duration.

### 3.3. Small Bowel Preparation

The global classification of bowel preparation, according to the Brotz scale was 8.2 ± 1.2 and 7.9 ± 1.0 points for the OMOM^®^ HD and Mirocam^®^ MC1600, respectively (*p* = 0.113; Table 2). However, when the cleanliness was evaluated per tertile, significant differences were obtained between the cases and controls. Overall, bowel preparation was rated better in the proximal tertile and when the OMOM^®^ HD device was applied. The mean scores for the first tertile were 8.5 ± 1.1 and 8.0 ± 1.1, while in the third mean ranks were 7.9 ± 1.6 and 7.1 ± 1.3 for cases and controls, respectively.

### 3.4. Small Bowel Capsule Endoscopy Findings

Relevant findings throughout the small bowel (DY) were detected in 118 patients, with no differences among the cases and controls (54.2 and 56.1%, respectively, *p* = 0.613). The most prevalent lesions were ulcers and erosions (detected in 31.8% of the patients), followed by angioectasia (18.2%); this pattern was similar despite the capsule system applied. Notably, SBCE was able to identify findings outside the small bowel in 13 patients (mostly gastric ulcers (n = 3), colonic angioectasia (n = 5), or colonic neoplasm (n = 1)).

## 4. Discussion

Over the past two decades, SBCE has established itself as an important tool in gastrointestinal examination. With an excellent safety record, SBCE devices have evolved technologically, broadening their capabilities in detecting small bowel pathology.

In this study, we compared two capsule endoscopy systems regarding visualization and diagnostic capacity. The aim was to provide evidence to support clinicians’ perception that more recent devices offer better image quality and to address the knowledge gap concerning the impact of imaging quality on diagnostic yield. The two devices under analysis, OMOM^®^ HD and Mirocam^®^ MC 1600, have never been formally compared before and belong to the same “average” price range Therefore, their comparison is timely not only from a clinical perspective but also from an economic point of view. Indeed, healthcare managers and clinicians may frequently encounter the need to decide among two “average-segment” devices, as “high-segment” systems (whose technical specificities would, at least theoretically, be superior), due to the scarcity of resources and the fair allocation principle [17].

In our cohort, the rate of complete small bowel examination was similar among models, and above the standard established by the European Society of Gastrointestinal Endoscopy (ESGE) performance measures (≥95%). The retention rate was below 1%, also within the target recommended by the ESGE (<2%).

Regarding small bowel preparation, we only found significant differences in the segmental analysis (per tertile), with the scores obtained from the OMOM^®^ HD group always being superior (*p* < 0.05). Considering that cases and controls followed the same preparation protocol (clear liquids on the previous day plus 4 L of KleanPrep) and were matched for factors known to impact bowel preparation (such as age and comorbidities [18]), it may be hypothesized that the differences are related to the characteristics of the device retrieving the images. Indeed, advanced optics may influence small bowel mucosal visualization [18]. In comparison to Mirocam^®^ MC1600, the OMOM^®^ HD system has a similar angle of view (172° versus 170°), fewer LED lights (four versus six), but a higher resolution (512 × 512 versus 320 × 320 pixels). Other relevant differences are that the OMOM^®^ HD device has an adaptative frame rate (2–10 frames per second (FPS)), while Mirocam^®^ MC1600 has a fixed frame rate of 6 FPS, and that the depth of field (DOF) is 0–30 mm and 0–50 mm for Mirocam^®^ MC1600 and OMOM^®^ HD, respectively [19]. A higher frame rate may reduce image blurring associated with the abrupt motion of the capsule [20]. On the other hand, the DOF corresponds to the distance between the nearest and furthest objects that are in focus in an image, or, in other words, the area of apparent sharpness. Conversely, both systems have adaptive brightness control algorithms, that adjust the exposure time depending on the incident light intensity of the sensor, avoiding both over-exposure and under-exposure scenarios. However, no complete information is available on these algorithms, hampering direct comparison among the models.

Another interesting finding was that the ranks of the first tertile were tendentiously higher. This brings into discussion two aspects. First, the segmental application of the Brotz scale (developed for global small bowel assessment) may be more informative. Indeed, providing a global rank may be misleading in cases where there are great asymmetries in cleanliness across different segments. This has been also highlighted by Macedo-Silva et al. [21], who developed a new segmental score (the SB-CLEAR, ranging from 0 to 9 points) and proposed that having ≤1 point in any segment should result in an inadequate overall classification. While we acknowledge that this new scoring system is advantageous, we were not able to use it due to the retrospective nature of our study. Second, the bowel preparation protocols in use appear to be worse in cleaning the distal small bowel. Whether this may compromise the detection of lesions in that segment remains unknown. Indeed, in a recent randomized controlled trial, no association was detected between the quality of the cleansing and the ability to detect significant lesions (P1 or P2 in the Saurin classification [22]); this is also in line with prior meta-analyses [9,23]. Further studies analyzing different preparation protocols, for example, those including boosters after the capsule has reached the small bowel [24,25], and evaluating segmental bowel preparation and diagnostic yield may clarify that issue.

In our cohort, the overall diagnostic yield was 55%, without significant differences between the two devices. This is in line with prior literature, that reported detection rates around 58% for all indications (ranging from 55% for OGIB to 66% for patients with diagnosed CD) [1]. Prior studies that compared different capsule systems (yet none evaluated Mirocam^®^ MC1600 versus OMOM^®^ HD) also failed to identify differences in diagnostic yield among different brands. In our study, the most common lesions were ulcers, erosions, and angioectasia. Similar lesions’ relative distribution have been reported in other cohorts enrolling patients with comparable indications [25]. 

The average small bowel transit time (SBTT) was within the previously described ranges [26,27]. However, it was significantly inferior in the group using the OMOM^®^ HD device (265 versus 307 min, *p* = 0.032). This finding may, more likely, be related to systematic differences in patients’ weight, height, or patterns of physical activity [28] that were not accounted for. However, it may also be hypothesized that it may be explained by the slightly different characteristics of the devices (OMOM^®^ HD capsule is 0.9 mm longer but 250 mg lighter than Mirocam^®^ MC1600), whose impact remains unknown. While some prior studies have reported that a longer SBTT correlated with higher diagnostic yields [29,30], others have found opposite associations. Indeed, in the study by Lasa et al. (2022) [22], the SBTT was significantly longer in patients with a bowel lesion (359 versus 279 min). In our study, no positive or negative associations were found. Indeed, the two devices had comparable percentages of positive findings, and no significant correlations between SBTT and DY were detected in our cohort (point biserial correlation 0.120, *p* = 0.080). Even though it seems that the OMOM^®^ HD capsule moved faster through the small bowel, intra- and inter-individual differences in SBTT may be a relevant confounding factor. Indeed, the intra-individual coefficient of variation among healthy controls has been reported to reach as high as 30% in prior studies [31].

In summary, even though the OMOM^®^ HD device offers better visualization (estimated through a validated cleanliness scale in two groups matched for potential confounders), both devices were safe, and there was no significant difference in their diagnostic yields. Even though this study was the first to compare OMOM^®^ HD and Mirocam^®^ MC1600 capsule endoscopy systems, this study has some limitations. First, its observational design may be prone to biases not amenable to statistical handling. Second, it was not possible to retrieve data regarding download duration. Indeed, the single study that compared OMOM^®^ HD with another system (from PillCam^®^) suggested that the main strength of the first was the significantly lower time for download [14]. In fact, the fast-downloading asset of OMOM^®^ HD, which allows data retrieval within 5 min, may be very relevant, particularly in an emergency setting. Third, no information was available concerning the time needed to examine the images, which may be very different among systems, considering the adaptative frame rate of OMOM^®^ HD but also the fact that the two software have different tools that eliminate repetitive frames (redundancy detection). Considering the busy clinical setting, having devices that allow better time (and cost)-effectiveness ratios is of utmost importance. Fourth, it was not possible to compare the ability to identify the Z-line or the ampulla of Vater, whose detection could be an indirect marker of possible missed lesions in the proximal small bowel [32], further validating the diagnostic yields.

## Figures and Tables

**Table 1:** Characteristics of the patients included in the study.

	Mirocam^®^ (before Matching, n = 306)	OMOM^®^ HD (n = 107)	Mirocam^®^ MC1600 (after Matching, n = 107)	*p* Value (Cases and Controls)
Age mean (SD)	48.4 (16.1)	54.0 (18.5)	52.9 (16.7)	0.751
Female % (n)	52.6 (161)	61.7 (66)	62.6 (67)	0.704
Karnofsky status-mean (SD)	90.3 (11.4)	97.1 (8.5)	95.9 (9.2)	0.689
Comorbidities n (%)				
Chronic heart disease	9.4 (29)	15.0 (16)	14.0 (15)	0.790
Diabetes mellitus	23.2 (71)	15.0 (16)	14.0 (15)	0.709
Chronic kidney disease	7.1 (22)	5.6 (6)	5.6 (6)	0.999
Chronic liver disease	3.9 (12)	4.7 (5)	3.7 (4)	0.658
Indication % (n)				
Iron-deficiency anemia	47.1 (144)	58.9 (63)	58.9 (63)	0.892
Overt bleeding	2.3 (7)	1.9 (2)	1.9 (2)	0.999
Suspected CD	30.7 (94)	26.2 (28)	27.1 (29)	0.708
Confirmed CD	18.9 (58)	11.2 (12)	10.3 (11)	0.617
Suspected SB tumor	1.0 (3)	1.9 (2)	1.9 (2)	0.999
Medication % (n)				
NSAID	24.5 (75)	20.6 (22)	21.4 (23)	0.728
SSRI	13.4 (41)	16.8 (18)	16.8 (18)	0.999
Metformin	19.0 (58)	9.3 (10)	8.4 (9)	0.830
Opioids	1.3 (4)	1.9 (2)	1.9 (2)	0.999

**Table 2:** Bowel preparation, transit times, and findings of the cases (OMOM^®^ HD) and controls (Mirocam^®^ MC1600).

Parameters		OMOM^®^ HD	Mirocam^®^ MC1600	*p* Value
Complete examination n (%)	-	104 (97.2)	105 (98.1)	*p* = 0.762
Capsule retention n (%)	-	1 (0.9)	1 (0.9)	*p* = 0.999
Transit times (min) mean (SD)	Gastric	53.0 (43.0)	56.0 (47.0)	*p* = 0.867
	Small bowel	265.0 (118.0)	307.0 (87.0)	*p* = 0.032
Bowel preparation ^1^ mean (SD)	Global small bowel	8.2 (1.2)	7.9 (1.0)	*p* = 0.113
	First tertile	8.5 (1.1)	8.0 (1.1)	*p* = 0.017
	Second tertile	8.2 (1.3)	7.5 (1.1)	*p* = 0.004
	Third tertile	7.9 (1.6)	7.1 (1.3)	*p* = 0.003
Findings % (n)	Diagnostic yield	54.2 (58)	56.1 (60)	*p* = 0.613
	Ulcers/erosions	30.8 (33)	32.7 (35)	*p* = 0.561
	Angioectasia	20.5 (22)	15.9 (17)	*p* = 0.103
	Sub-epithelial lesion	2.8 (3)	0.9 (1)	*p* = 0.099
	Adenocarcinoma	0.9 (1)	0.0 (0)	*p* = 0.394
	Extra-small bowel	6.1 (7)	5.6 (6)	*p* = 0.112

^1^ Classified according to the adapted Brotz scale.

## Data Availability

The data underlying this article can be shared upon reasonable request to the corresponding author.
